# Platform-Mediated Visibility of Exploitation Indicators: A Cross-Platform Analysis of AdultWork (UK) and CallEscort (US)

**DOI:** 10.3390/bs16091591

**Published:** 2026-09-07

**Authors:** Arianna Barbin, Gregory J. Bott, Nickolas K. Freeman, Sarah Hambidge

**Affiliations:** 1 School of Psychology, Faculty of Media, Science, & Technology, Bournemouth University, Poole BH12 5BB, UK; 2 Department of Information Systems, Statistics, and Management Science, Culverhouse College of Business, The University of Alabama, Tuscaloosa, AL 35487, USAfreem028@ua.edu (N.K.F.)

**Keywords:** trafficking for sexual exploitation, online commercial sex, coordinated activity, platform architecture, technology-facilitated gender-based violence

## Abstract

Online commercial sex platforms have become key environments in which potential indicators relevant to technology-facilitated sexual exploitation (TFSE) and technology-facilitated gender-based violence (TFGBV) may be observed. Current language models and computational approaches to identifying potential exploitation often assume that behavioural indicators (e.g., geographical mobility, pricing, and other linguistic markers) are easily accessible and comparable across online spaces and jurisdictions. This study challenges this assumption by showing that platform design largely shapes the variables and indicators that can be observed in these spaces. Drawing on a large-scale dataset of 1.9 million profiles (after cleaning) from two major online commercial-sex platforms, AdultWork (United Kingdom) and CallEscort (United States), the research examines how potential exploitation-related indicators may be identified and interpreted across platform architectures. Through cross-platform mapping, significant misalignments in data visibility and comparability were highlighted for demographic characteristics, mobility data, and services offered. To address comparability challenges, the study introduces a platform-mediated visibility framework to explain how platform design shapes the visibility, interpretation, and comparability of potential indicators relevant to TFSE and TFGBV. The framework provides a methodological basis for interpreting exploitation-related indicators across different platform architectures and underscores the value of platform-aware approaches to safeguarding and prevention. In collaboration with US and UK law enforcement and Non-Governmental Oranization (NGO) partners, the study advocates for proactive and tailored prevention strategies that account for platform architecture while recognising that coordinated activity and potential indicators do not, in isolation, establish exploitation, trafficking or criminal behaviour.

## 1. Introduction

Technology-facilitated sexual exploitation (TFSE) can be understood within the broader framework of technology-facilitated gender-based violence (TFGBV), involving the use of digital technologies to recruit, groom, coerce, control, advertise, and exploit individuals for sexual purposes (Henry & Powell, 2018; Sheikh & Rogers, 2024). As these activities increasingly occur through digital platforms, they generate digital traces that may provide opportunities for the detection and prevention of exploitation (N. K. Freeman et al., 2022). Sex trafficking represents a prominent form of TFSE, disproportionately affecting women and girls, and causing significant physical, psychological, social, and economic harm (Deshpande & Nour, 2013; International Labour Organization et al., 2022). The growing integration of social media, online advertising platforms, digital communication technologies, and online payment systems into trafficking operations has transformed how exploitation is organised and sustained across geographical boundaries while simultaneously generating digital traces that may support detection and prevention efforts (N. K. Freeman et al., 2022; Gerry & Shaw, 2019; Latonero, 2011). From both a legal and ethical perspective, online platforms hosting commercial sex advertisements occupy a complex digital space (Swords et al., 2021). These platforms can provide sex workers with tools for independent advertising, client screening, and income generation (Cunningham et al., 2018). However, they are also used by traffickers to recruit, advertise, monitor, and profit from vulnerable victims (Cowen & Colosi, 2021; N. K. Freeman et al., 2022; Latonero, 2011; Rossy & Ribaux, 2020). As a result of this duality, TFSE and consensual sex work can coexist within the same digital environments, making it often difficult to distinguish one from the other (Döring et al., 2022; Pajnik & Kuhar, 2025). Understanding how exploitation exists within digital environments has become increasingly important for informing prevention and detection efforts (Stephenson et al., 2025).

Although trafficking has long been recognised as a transnational crime, evidence suggests that trafficking patterns and victimisation experiences vary according to national contexts, including differences in legislative frameworks, migration patterns, labour market demands, and enforcement practices (Cockbain & Bowers, 2019). The United Kingdom (UK) and the United States (US) represent two high-priority jurisdictions for TFSE research and intervention. The UK is a destination and transit country for victims trafficked from Europe, Africa, and Asia (Antonopoulos et al., 2019), while the US shows important distinctions between domestic and international trafficking networks in terms of victim profiles, recruitment practices, and operational structures (Veldhuizen-Ochodničanová et al., 2020). Both jurisdictions have witnessed the migration of sex work advertising from classified newspaper listings and street-level contact to profile-based digital platforms, a shift that has created new opportunities for researchers and law enforcement but also introduced new methodological challenges (Hester et al., 2019; Sanders & Keighley, 2023). Comparing indicators across platforms has therefore become essential for determining whether potential indicators of exploitation are consistent across different platform architectures, and whether approaches developed for one online commercial-sex platform transfer to others (Cockbain & Bowers, 2019; Giommoni & Ikwu, 2024). Examining major online escort platforms is one tractable method for generating such insight, though it is bounded by each platform’s structure and reach; because platform and national market are closely intertwined here, observed differences and similarities are best read as platform-level rather than national. Little attention has also been given to whether patterns of coordinated activity emerge across large-scale platform data or how such patterns should be interpreted without assuming that they represent coordinated exploitation networks (N. K. Freeman et al., 2022; Meyer & Shelley, 2020).

Despite growing interest in TFSE, existing research has largely examined potential trafficking indicators within specific platforms, datasets, or national contexts, with much of the evidence derived from single-jurisdiction studies and platform-specific analyses (Gezinski & Gonzalez-Pons, 2024; Giommoni & Ikwu, 2024; Ibanez & Suthers, 2014). A fundamental but underexamined assumption underpins most of this published work: that potential trafficking indicators are stable, directly observable properties of user behaviour that can be read from platform data (de Vries & Cockbain, 2024). However, online commercial-sex platform design choices (e.g., what information to elicit, how to structure it, and what to make machine-readable) may contribute to determining which behavioural signals become visible as data and which remain obscured or require reconstruction (Koenig et al., 2022). Differences in platform architecture may produce variation in apparent indicator prevalence that reflects structural encoding rather than differences in underlying behaviour, with direct consequences for the validity of cross-platform and cross-national comparisons (Gillespie, 2018; van Dijck, 2014). However, this has not yet been tested across online commercial-sex platforms. This is an important gap in TFGBV research, as computational methods can only detect what platforms render detectable, and their outputs are therefore shaped as much by infrastructural conditions as by the behaviours and potential exploitation-related indicators being examined (Plantin & de Seta, 2019).

### 1.1. Existing Approaches to Identifying Potential TFSE Indicators in Online Environments

Researchers have developed a range of approaches to identify potential indicators of TFSE within online environments, drawing upon data generated through social media platforms, online communication technologies, commercial sex advertising websites, and other digital ecosystems that increasingly feature within broader discussions of TFGBV (Latonero, 2011). The literature focuses, for example, on identifying potential individual trafficking indicators within advertisements or user profiles, including specific language patterns, references to youth, movement between locations, shared contact details, and indicators of coercion or third-party control (Ibanez & Suthers, 2014; Polaris Project, 2017). These indicators were largely derived from practitioner knowledge and qualitative analysis of confirmed trafficking cases, then operationalised as features to be searched for in large advertisement datasets.

The underlying assumption that trafficking leaves identifiable digital traces, including linguistic and behavioural indicators proposed to distinguish trafficking from consensual sex work, remains influential but has attracted sustained methodological critique. Giommoni (2024) demonstrated that no published indicator framework can achieve sufficient precision for individual-level victim identification, while empirical analysis of UK online sex market data found that indicators such as geographic mobility, templated language, and below-market pricing frequently occur within consensual sex work as well as exploitation, producing substantial false-positive rates when applied to unverified datasets (Giommoni & Ikwu, 2024). Lugo-Graulich et al. (2024) similarly found that single indicators provide weak discriminatory power in isolation, and that temporal and co-occurrence patterns offer stronger, though still imperfect, signalling. More recently, large-scale computational approaches have extended trafficking detection beyond individual advertisement analysis. Dubrawski et al. (2015) highlighted that connecting profiles through shared contact information using network analysis substantially outperforms single-indicator screening. Summers et al. (2023) showed that combining multiple structural features in a multi-input classification model, including posting frequency, text similarity, pricing patterns, and geographic variables, achieved higher discriminative accuracy than any single feature in isolation. Keskin et al. (2021) applied operational research methods to identify movement patterns and network structures in trafficking datasets, while N. K. Freeman et al. (2022) described how collaboration between researchers and law enforcement, using digital trace data from online sex markets, could support active disruption of trafficking networks. Lastly, Tamarit-Sumalla et al. (2025) identified an association between online sex adverts in Spain and content-based indicators of technology-facilitated victimisation. Overall, these studies suggest that relational and network-level analyses, which treat profiles as interconnected nodes within a wider network, may provide a basis for identifying coordinated activity and potential exploitation-related indicators in online environments. In addition, previous research has also proposed a range of linguistic indicators of exploitation, including duplicated or templated profile text, third-person or managerial language, youth-coded terminology, and descriptions suggestive of transient or circuit-based mobility (Dubrawski et al., 2015; Portnoff et al., 2017; Polaris Project, 2017; Lugo-Graulich et al., 2024). As with behavioural indicators, the visibility and interpretation of these linguistic features are likely to be shaped by platform architecture and the context in which they appear.

Despite this, significant challenges remain. Many studies have focused on individual advertisements, specific datasets, or single-jurisdiction investigations, limiting the understanding of whether indicators identified in one context are transferable to other platforms and jurisdictions (Giommoni & Ikwu, 2024). These challenges are further compounded by restricted access to platform data, the dynamic nature of online commercial sex markets, and ethical concerns surrounding privacy, consent, and the identification of potentially vulnerable individuals (Benítez-Hidalgo et al., 2026; University of Melbourne & United Nations Population Fund, 2023).

### 1.2. The Current Study

The present study examines whether patterns of coordinated activity are evident across large-scale advertising data from two commercial sex platforms with contrasting architectures (AdultWork and CallEscort), and how these patterns should be interpreted in relation to potential TFSE indicators.

Rather than attempting to determine whether individual users are victims of trafficking or TFGBV, the study analyses profile data from two major online commercial sex advertising platforms operating in the UK and the US. By comparing account characteristics across jurisdictions, the study contributes to understanding how platform architecture shapes the visibility and interpretation of potential exploitation-related indicators. Beyond this comparative analysis, the findings aim to support the development of scalable research approaches for examining potential indicators of TFSE and TFGBV in online environments.

Expanding on previous work, the following research questions are addressed:

RQ1. How do the structure, availability, and encoding of profile data differ between the two platform architectures, and what are the implications for the interpretation of potential TFSE indicators?
RQ2. Can profile-level analysis of online commercial sex work platforms identify patterns consistent with TFSE indicators, and to what extent does platform architecture constrain that identification?
RQ3. Can profile-level differences be used to inform a Platform-Mediated Visibility Framework?

## 2. Methods

### 2.1. Dataset

The current dataset draws from a larger, ongoing collection effort spanning multiple online commercial sex platforms across the UK and the US. The present analysis focuses on two platforms selected for comparative cross-platform analysis: AdultWork (UK) and CallEscort (US). We do not treat AdultWork (UK) and CallEscort (US) as full proxies for their respective national markets. Our comparison is primarily architectural and centres on how each platform structures and exposes profile information. Note that the two platforms nonetheless operate within (and are likely shaped by) divergent legal environments: the sale of sex is broadly lawful in England and Wales, whereas it remains criminalised in nearly all of the United States^1^. This contrast provides interpretive context for the architectural differences we observe, although it does not imply that either site represents its national market as a whole.

We selected these two platforms for four reasons: (1) each is the highest-volume profile site in its national market; (2) both maintain sufficiently structured or semi-structured profile architectures to permit systematic data extraction; (3) together they support cross-national comparison; and (4) the law enforcement and non-governmental organisation (NGO) partners engaged in this research identified both platforms as operationally relevant to TFSE investigations. Both platforms are also profile-based sites that host accounts advertising commercial sex services, distinguishing them from ad-based platforms that have been the predominant focus of prior trafficking research (Giommoni & Ikwu, 2024; Lugo-Graulich et al., 2024; Summers et al., 2023). Unlike ad-based data, profiles generate longitudinal records of profile activity, including service offerings, pricing histories, client reviews, and geographic movement patterns over time (Kjellgren, 2024). Importantly, the profile-based architecture of these specific platforms enables the capture of longitudinal behavioural data beyond individual advertisements, allowing researchers to examine broader patterns of connection and coordination through which digitally mediated sexual services are advertised and monitored (N. Freeman et al., 2026). Collectively, these characteristics make profile-based data well-suited to future community detection and network identification approaches in ways that were not prioritised for this manuscript (Sperlì, 2025); the present study establishes, nonetheless, the indicator-visibility foundation on which such analyses could be built.

Unlike profile-based platforms, ad repositories primarily archive advertisements and campaign metadata rather than continuous user-level behavioural data, making them less suited to longitudinal coordination analysis of user-level behaviours (Santini et al., 2026). The resulting AdultWork dataset comprises 24,262 user profiles collected between February and March 2026, supplemented by 318,758 reviews, 575,738 service listings, and 87,608 pricing records. The CallEscort dataset comprises 2,267,134 profiles collected between April 2022 and April 2026, with 114,730 associated reviews. The difference in timing for data collection is addressed through the equalisation procedure described in Section 2.3 and is further discussed in Section 5.

### 2.2. Ethics and Data Protection

The study received ethical approval from [redacted for peer review]. It analysed data collected from publicly accessible profile pages on AdultWork (UK) and CallEscort (US) and did not involve direct interaction with individuals. Despite this, public accessibility was not treated as removing the ethical interests or reasonable privacy expectations of the individuals represented. Particular consideration was given to the sensitivity of commercial-sex data, the potential vulnerability of individuals experiencing trafficking for sexual exploitation (TFSE) or other harms, and the consequences of identification or misclassification.

Identifying information, including contact details, was excluded or transformed during data processing wherever possible. Access to raw profile-level data was restricted to the researchers responsible for data collection and processing, and findings are reported only in aggregated form or in a form that does not identify individual profiles. Profile images were blurred before analysis by the wider research team, with original images accessible only to those responsible for data collection and processing. This approach was consistent with Giommoni and Ikwu (2024) and formed part of the measures used to support responsible digital research.

The present study is exploratory and does not produce or validate an operational detection tool. Apparent coordination does not establish trafficking, coercion, exploitation, or criminal conduct, and the approach cannot reliably distinguish consensual commercial-sex activity from exploitation. Such patterns may instead reflect consensual working arrangements, shared premises, collective safety practices, administrative support, or common advertising conventions. They should therefore be treated only as potential indicators requiring contextual assessment and corroboration, rather than as evidence of TFSE or criminality. The findings are not intended to support automated enforcement decisions, the targeting of individual sex workers, or intervention based solely on the patterns identified. Any future operational application would require further validation, independent corroboration, meaningful human review, assessment of necessity, proportionality and potential discriminatory effects, and explicit consideration of the risk of surveilling, exposing or criminalising consensual sex workers. Any such development should also involve engagement with sex workers, survivors, and relevant specialist organisations.

### 2.3. Procedure

Data were collected using custom Python-based web scrapers developed separately for each platform, drawing on publicly accessible profile pages/c. Data were scraped from two platforms: CallEscort (*n* = 2,267,134 profiles; 21 April 2022 to 29 April 2026) and AdultWork (*n* = 24,262 profiles; 10 February 2026 to 31 March 2026), capturing profile updates, new listings, and changes to existing records across the respective collection windows. Data were stored in a relational database structured across four linked tables: a primary profiles table and subsidiary tables capturing reviews, services, and pricing records, each connected through a unified profile identifier. Pre-processing followed four stages. First, duplicate records arising from repeated scraping cycles were identified and removed. Second, profiles meeting predefined criteria for incompleteness or potential automation were removed. Profiles missing a description together with their platform’s primary identifying field—city on AdultWork, ad headline on CallEscort, since CallEscort populates location fields by construction—were excluded as incomplete (1541 AdultWork, 6.4%; 7935 CallEscort, 0.4%). Following stage 2, 1541 AdultWork profiles (6.4%) and 7935 CallEscort profiles (0.4%) were removed. Five further criteria were applied to identify profiles that were potentially automated or contained insufficient information for analysis: (a) description text appearing identically across ten or more distinct profiles (AdultWork: 933 removed; CallEscort: 330,716 removed); (b) recognisable cam-service template text on AdultWork (779 online camera show-only profiles removed, AdultWork only); (c) zero images, zero reviews, and zero listed services simultaneously (AdultWork: 2977; CallEscort: 8890); (d) phone numbers shared across three or more distinct provider identifiers (AdultWork: 52; CallEscort: 1176 records showed repeated posting linked to the same account); and (e) descriptions of twenty characters or fewer (AdultWork: 66; CallEscort: 25,466). Profiles meeting any single criterion were excluded. Table 1 summarises the filtering outcomes; the cleaned datasets comprise 19,488 AdultWork profiles and 1,909,361 CallEscort profiles. A subsequent equalisation step, described below, produced the final analytic sample of 17,061 profiles per platform. Third, service classifications on AdultWork were normalised from raw platform labels into a standardised taxonomy. Additionally, clientele preference fields were harmonised across both platforms by parsing free-text values into structured Boolean indicators (e.g., accepts_men, accepts_women, accepts_couples, accepts_nonbinary). Fourth, the structural asymmetry between the two platforms was addressed directly: while AdultWork organises user information through discrete, machine-readable metadata fields, CallEscort embeds comparable information within free-text narrative descriptions. The ‘before down-sampling’ row reflects the output of the cleaning stages only; the equalised analytic sample (*n* = 17,061 per platform) is described in the text following Table 1.

The cleaned datasets differed by approximately two orders of magnitude in scale (19,488 AdultWork profiles versus 1,909,361 CallEscort profiles) and by approximately 30-fold in collection window (10 February–31 March 2026 for AdultWork; 21 April 2022–29 April 2026 for CallEscort). Raw counts and rates were therefore not directly comparable across platforms. To support the matched cross-sectional comparison specified in RQ2, the following equalisation procedure was applied to the cleaned data.

First, the CallEscort dataset was restricted to the AdultWork collection window (10 February–31 March 2026), yielding 66,769 advertisements. Second, advertisements were deduplicated by phone number, retaining the most recent scrape for each unique number, producing 66,744 unique advertisers (one row per advertiser). Third, a stratified random subsample was drawn from these 66,744 advertisers. Strata were defined as the cross-product of US state and scrape-month (102 strata in total). Allocation within strata was proportional to each stratum’s share of the eligible pool, with largest-remainder rounding applied to ensure integer counts summing exactly to the target. The random seed was 564. The resulting CallEscort analytic sample comprised 17,061 advertisers.

Fourth, the AdultWork dataset was restricted to profiles identified as women, yielding 17,061 profiles. This restriction was necessary because CallEscort does not record gender as a structured field; restricting AdultWork to women is the closest available alignment, and the gender variable in the analytic sample is therefore 100% by design rather than by selection.

The final analytic sample thus comprises 17,061 profiles per platform (*n* = 34,122 in total), drawn from the same seven-week window (10 February–31 March 2026). No rows were deleted from the underlying database: membership in the analytic sample is flagged by the field ads.analytic_sample = 1, and the associated ads.sample_weight variable (weights summing to 66,744 for CallEscort) permits recovery of full-window estimates for any variable of interest.

Balance checks confirmed that the subsampled CallEscort distribution was representative of the eligible pool. All standardised mean differences between the sample and the eligible pool were below 0.01 in absolute value. State-level shares differed by no more than 0.005 percentage points, and month-level shares by no more than 0.006 percentage points. One hundred bootstrap redraws of the stratified sample yielded a mean CallEscort age of 27.241 years (SD = 0.038, range 27.14–27.34), centring on the eligible-pool mean of 27.240. The achieved analytic sample’s mean of 27.261 (reported as 27.3 in Table 2) lies within 0.52 SD of the re-draw mean and inside the re-draw range, so the small difference between the bootstrap value (rounded to 27.2) and the Table 2 value (27.3) reflects expected sampling variation rather than an inconsistency.

This design supports a matched cross-sectional comparison of platform-level characteristics and indicator prevalence under equivalent sampling conditions. It does not support longitudinal or temporal-trend analysis: such analyses remain restricted to the US platform and are conducted on the full CallEscort dataset (1,909,361 profiles, April 2022–April 2026) outside the equalised sample.

Profiles may match more than one criterion; the total reflects distinct profiles excluded under any criterion. We selected the ≥10 duplicate-description threshold to target high-confidence automated posting while minimising false positives among profiles using similar template language.

### 2.4. Analysis

The analysis was designed to identify patterns of coordinated activity within platform ecosystems rather than determine whether the individuals represented were experiencing trafficking or exploitation. Instead, findings are interpreted as descriptive patterns of coordinated activity that require contextual assessment and may inform future TFSE research and prevention efforts. The analysis draws on the analytical framework previously developed by Freeman and colleagues for examining online commercial sex ecosystems (N. K. Freeman et al., 2022; N. Freeman et al., 2025, 2026). Rather than analysing profiles individually, this framework conceptualises online commercial sex platforms as ecosystems of interconnected accounts, enabling the identification of patterns of coordinated activity that may not be observable at the level of individual advertisements or profiles. The present study extends this framework through a comparative analysis of profile-based commercial sex platforms operating in the UK and the US, applying its profile-level, descriptive component, while the network-level and community-detection components remain the subject of ongoing and future work. To address RQ1, comparative analyses examined differences in the structure, availability, and encoding of profile data between the AdultWork and CallEscort datasets, identifying which variables are represented as discrete fields, embedded within narrative text, or absent altogether. To address RQ2, profile-level data were examined to establish which patterns, consistent with previously identified TFSE indicators (Keskin et al., 2021; N. K. Freeman et al., 2022), remain identifiable on each platform, and how platform architecture constrains identification. To address RQ3, the structural and encoding differences documented under RQ1 and RQ2 were synthesised into the Platform-Mediated Visibility Framework presented in the Discussion.

## 3. Results

### 3.1. Descriptive Characteristics

Table 2 presents descriptive characteristics of the two datasets. The most pronounced difference is in age distribution. The mean age of profiles on AdultWork is 33.9 years (SD = 9.7), compared with 27.3 years (SD = 5.9) on CallEscort; a difference of 6.7 years (computed from the unrounded means of 33.944 and 27.261).

This is reflected in the age-group breakdown: on CallEscort, 34.6% of profiles with available age data fall within the 18–24 range and a further 54.1% in the 25–34 range, meaning 88.7% of profiles with available age data are under 35 years old. On AdultWork, the distribution is substantially older and flatter, with only 13.1% in the 18–24 group and 24.2% in the 35–44 group. Moreover, on AdultWork, age is captured as an explicit, machine-readable metadata field, with data available for 78.7% of profiles. On CallEscort, there is no equivalent structured age field; age is inferred, instead, from profile text or category metadata and is available for 99.9% of profiles (*n* = 17,055). The 21.2 percentage-point gap in age-data availability (CallEscort 99.9% vs. AdultWork 78.7%) is in itself a structural finding: it reflects platform design choices about what information to elicit and how to encode it internally. Similarly, gender, sexual orientation, as well as nationality data are available as structured variables only for AdultWork; CallEscort does not store equivalent structured fields, rendering these dimensions analytically inaccessible without NLP extraction. Geographic concentration patterns differ across jurisdictions: UK AdultWork platform profiles concentrate in major cities (London, Manchester, Birmingham), consistent with known domestic sex work market structures, while US CallEscort platform profiles show more even distribution across a larger number of metropolitan centres.

### 3.2. Unmatched Fields Across AdultWork and CallEscort

To assess comparability across platforms, variables from both datasets were systematically mapped considering the platform descriptors. The comparative examination of unmatched variables between the UK-based AdultWork dataset and the US-based CallEscort dataset reveals substantial differences in platform architecture, data normalisation, and information representation relevant to potential TFSE-related behavioural indicators.

Table 3 summarises these findings.

Although several variables could not be directly matched across platforms, several nevertheless captured conceptually similar forms of information through different structural mechanisms. Within the context of TFGBV, these distinctions are significant because platform design directly shapes how mobility, commercial sexual activity, and potential indicators relevant to exploitation or coercion become visible, concealed, standardised, or operationalised across platform architectures within digital environments (Summers et al., 2023).

The comparison shows that AdultWork adopts a highly structured platform architecture in which demographic information, services, pricing, and profile metadata are represented through discrete machine-readable variables. Service offerings are stored through standardised categorical fields, while pricing is listed as separate variables (e.g., pricing.incall; pricing.outcall). Demographic characteristics, including gender, nationality, ethnicity, and sexual orientation, are also explicitly represented through structured metadata. Moreover, AdultWork incorporates extensive profile-level attributes relating to physical appearance, account activity, verification status, and user engagement. The platform further extends this structured architecture through interview-style profile prompts, review systems, categorised service taxonomies, and customisable profile sections. Conversely, CallEscort relies more heavily on unstructured narrative advertisement text to communicate relatively similar information. Services, pricing, nationality descriptors, and potential behavioural indicators are commonly embedded within free-text descriptions rather than represented as standardised categorical variables. Pricing may appear through abbreviated conventions such as “250QV” or “475HR”, while nationality and ethnicity can only be inferred from descriptive phrases embedded within advertisement narratives. Gender is similarly implied through site-level categorisation structures rather than explicitly captured within metadata fields.

A further divergence was observed in the representation of geographic mobility. AdultWork captures travel behaviour through interview-style prompts asking users to list “Towns/Areas you will visit,” whereas CallEscort operationalises mobility through “Cities worked” variables or city-count measures. Although structurally distinct, both approaches capture forms of geographic circulation commonly examined in trafficking research and the movement dynamics of online commercial sex markets (N. K. Freeman et al., 2022; Kjellgren, 2024; Giommoni & Ikwu, 2024). Importantly, the different representations of mobility also reflect differing platform expectations regarding self-disclosure and advertiser visibility.

The comparison also identified several CallEscort variables with limited analytical value due to redundancy or semantic inconsistency. The “Currently in field” label, for example, largely duplicated information already represented through matched city and state variables, while the location field frequently contained contact details or promotional material rather than meaningful geographic information, which could have aided analysis. These inconsistencies further illustrate how loosely structured platforms can complicate attempts to identify reliable potential exploitation-related indicators. Together, these findings reveal a substantial structural asymmetry between the two datasets, impacting the visibility and consistency of potential TFSE-related indicators across platforms. AdultWork’s normalised schema facilitates direct quantitative analysis and standardised feature extraction, while CallEscort provides only a subset of the structural attributes provided by AdultWork. More extensive NLP operations are required to reconstruct equivalent indicators from unstructured text (Diaz & Panangadan, 2020; Hein et al., 2025). Indicators that appear highly prevalent within AdultWork may reflect the platform’s greater degree of standardisation rather than substantive behavioural differences, whereas equivalent indicators within CallEscort may remain partially obscured within narrative text and become underrepresented during extraction and analysis.

### 3.3. Language and Content Indicators

Previous research has interpreted templated or substantially duplicated profile text appearing across multiple identities, locations, or time periods as a potential indicator of centralised authorship by a third party rather than independent profile management (Dubrawski et al., 2015; Portnoff et al., 2017). Third-person or managerial voice (phrases such as ‘she is available,’ ‘contact our agency,’ or ‘book through us’) has similarly been identified as a potential indicator of intermediary involvement or control (Polaris Project, 2017). Youth-coded vocabulary, including terms such as ‘new,’ ‘fresh,’ ‘new to the scene,’ or ‘barely legal,’ has been examined as a potential language-based indicator of the exploitation of minors (Lugo-Graulich et al., 2024). Short-stay language signalling geographic impermanence (‘in town for a few days,’ ‘just visiting,’ ‘leaving soon’) has been proposed as a potential textual indicator of circuit mobility (Polaris Project, 2017). The pre-processing stage identified 933 AdultWork profiles and 330,716 CallEscort profiles sharing text-identical descriptions across 10 or more distinct profiles; a rate of 3.8% and 14.6%, respectively. While these profiles were excluded from the analytic dataset as likely automated, the scale of duplication on CallEscort (more than 330,000 profiles sharing non-unique text) is noteworthy and indicates extensive template reuse, although this cannot establish centralised or third-party profile management. The higher absolute count on CallEscort reflects dataset size; the rates are very low on both platforms (0.2% for AdultWork and 0.1% for CallEscort), with AdultWork’s rate approximately twice that of CallEscort.

### 3.4. Geographic Mobility Indicators

Reuse of the same contact information across profiles appearing in multiple cities constitutes a core signal in phone-network analysis (Dubrawski et al., 2015). Area code mismatches (e.g., where a posted phone number does not correspond to the advertised location) provide an additional structural indicator detectable without text analysis. Temporal clustering of profile activity around major public events has been associated with trafficking demand patterns (Polaris Project, 2017), as have sequential city appearance patterns consistent with known trafficking circuits. The geographic and touring variables within the AdultWork dataset, including but not limited to the explicit touring flags and travel destination fields, map directly onto this indicator category. Pre-processing identified 52 AdultWork profiles meeting the shared-phone criterion (intended as any phone number shared across three or more distinct provider identifiers). Additionally, 1176 records on CallEscort were flagged as associated with repeated posting from the same account. To account for this difference, the CallEscort count was treated as a descriptive identifier of repeated posting, as opposed to evidence of shared contact across similar ads. The AdultWork shared-number signal has been considered in prior network-analytic work (Dubrawski et al., 2015; N. K. Freeman et al., 2022), which documented the challenges in independently establishing whether a third-party control or exploitation is occurring. These challenges have been associated with posting practices and advertisements that can also be part of consensual arrangements, shared premises, administrative support, or collective working practices among posting individuals (N. K. Freeman et al., 2022). For this variable, although not identical in terms of what they measure, the corresponding rates have been reported in Table 1 and should not be treated as directly comparable.

### 3.5. Structural and Pricing Indicators

Pricing and profile management characteristics have also been identified in previous research as potential indicators of exploitation (Tamarit-Sumalla et al., 2025). For instance, extremely low charges (below-market pricing), particularly when services are offered in very limited time slots, have been associated with high-volume models that have been examined as potential TFSE- related indicators (Dubrawski et al., 2015; Polaris Project, 2017). Research and legislation have, in fact, argued that this may indicate situations where individuals are coerced to work under pressure to meet financial targets or quotas forcibly set by others (e.g., traffickers), limiting control over autonomous and legal working conditions (Crown Prosecution Service, 2025). Similarly, in-platform availability framing, such as ‘24/7 available’ or ‘always in’ may also imply limited agency over scheduling and working hours (Lugo-Graulich et al., 2024). References to third-party booking, or the presence of multiple distinct profile identities sharing a single contact channel, have been proposed as potential indicators of shared management or third-party control across both structured and unstructured platform data (Portnoff et al., 2017). The pricing variables in the AdultWork dataset (including hourly and session-rate data) enable systematic pricing-pattern analysis. On CallEscort, pricing is embedded in narrative text and must be extracted before comparable analysis is possible. This means that pricing-based signals are directly quantifiable for one jurisdiction and require reconstruction for the other; a limitation that must be accounted for in any cross-platform comparison of potential exploitation-related pricing indicators. Examining whether pricing patterns are associated with exploitation was beyond the scope of this study; instead, the analysis focused on how platform architecture shapes the visibility and comparability of pricing-related data.

### 3.6. Network-Level Patterns

At the network and computational level, multi-indicator and graph-based methods have extended trafficking detection beyond individual profile analysis. As discussed in the Introduction, these multi-indicator and network-based approaches substantially outperform single-indicator screening. Lugo-Graulich et al. (2024) similarly identified that temporal and co-occurrence patterns provided a stronger signal than individual features. The availability of structured, longitudinal profile data across both platforms, including review histories, pricing records, and service listings linked to persistent profile identifiers, positions this dataset as a resource for the kind of multi-indicator network analysis these studies recommend. While community detection and full network identification analyses will likely be conducted in the future, the present findings provided an essential methodological foundation for subsequent analyses by establishing which indicators are available, in what form, and with what analytical implications across these two specific platforms (AdultWork and CallEscort).

## 4. Discussion

This study examined how potential indicators associated with TFSE are represented across two online commercial sex platforms operating in fundamentally different architectural designs. Existing research has largely focused on identifying potential trafficking indicators within online advertisements and profiles, including geographic mobility, pricing patterns, textual markers, shared contact information, and indicators of third-party control (Dubrawski et al., 2015; Kjellgren, 2024; Polaris Project, 2017; Portnoff et al., 2017). More recent work has increasingly employed computational methods to combine multiple indicators through machine learning, network analysis, and large-scale digital trace analysis (N. K. Freeman et al., 2022; Graham et al., 2024; Lugo-Graulich et al., 2024; Summers et al., 2023). Across this literature, however, there is a common assumption that indicators are observable and available for analysis. The findings presented here suggest that this assumption warrants closer scrutiny.

The comparative analysis revealed that conceptually similar forms of information relating to demographics, services, pricing, mobility, and profile activity were present across both datasets but were represented through fundamentally different architectural arrangements. AdultWork captured many attributes through structured metadata fields, whereas CallEscort embedded equivalent information within narrative profile text. Similarly to what was previously identified for child sexual exploitation research (Lu et al., 2024), potential indicators associated with exploitation were not uniformly visible across the two platforms. Information that could be directly extracted and analysed within one platform often required substantial reconstruction through natural language processing and entity extraction techniques within the other. The issue is therefore not solely whether indicators are predictive of exploitation, but whether they are observable in the first place. This finding extends existing critiques of trafficking indicator frameworks. These precision limitations are discussed further below. Indicators such as mobility, advertisement similarity, or below-market pricing may characterise both consensual sex work and exploitative situations.

A critical methodological caveat governs the interpretation of the indicators examined here, before the study’s contribution is presented. Consistent with previous research, it is not possible to reliably identify a trafficking victim from online profile data in isolation (Sanders & Keighley, 2023). Giommoni (2024), most notably, already highlighted that no published indicator framework can yet achieve sufficient precision for individual-level classification, and empirical analysis of UK online sex market data found that applying standard indicator sets produced substantial false-positive rates among consensual sex workers (Giommoni & Ikwu, 2024). Independent sex workers may exhibit many of the same profile features attributed to trafficking, including geographic mobility, pricing structures, and multi-platform presence, without any third-party coercion (Kjellgren, 2024; L’Hoiry et al., 2024). Accordingly, this study treats indicators as potentially relevant patterns to be examined at an aggregate or network-level, rather than as evidence supporting inferences about individual profiles.

The present study additionally identifies an earlier analytical problem that precedes questions of predictive validity. Indicators cannot contribute meaningfully to detection where they are absent, inconsistently represented, or encoded in forms that are inaccessible to analysis (Saner et al., 2018; Bouché et al., 2026). Visibility was, therefore, identified here as a precondition for validity. This may help explain why indicators identified within one platform environment may not transfer effectively to another, despite describing ostensibly similar behaviours. It was through this analysis, and integration with the literature, that the authors here developed the Platform-Mediated Visibility Framework (PMVF) (RQ3). Conceptually, the PMVF draws on three theoretical concepts. First, the sociology of datafication (van Dijck, 2014; Kitchin, 2014), which establishes that data are not pre-given facts, but artefacts produced through technical infrastructure, classification systems, and representational choices. Second, platform affordance theory (Bucher & Helmond, 2018; Gillespie, 2018), which emphasises that platforms do not passively host activity but actively shape what users can do and what becomes recordable. Third, the epistemic infrastructure literature (Bowker & Star, 1999; Edwards et al., 2011), which shows how the standards, classifications, and data models embedded in technical systems determine what kinds of knowledge are producible from them. Together, these theories support the PMVF’s central claim that underlying patterns, behaviours, and parameters need to be contextualised within platform characteristics and barriers. For the scope of this analysis, PMVF was applied to sexual exploitation to raise the criticism that what becomes observable in TFSE research may not be a direct reflection of exploitative behaviour but an outcome of how platforms configure, encode, and surface user activity into data; and that any TFSE or TFGBV research design overlooking this risks fundamental validity threats.

**Figure 1:**
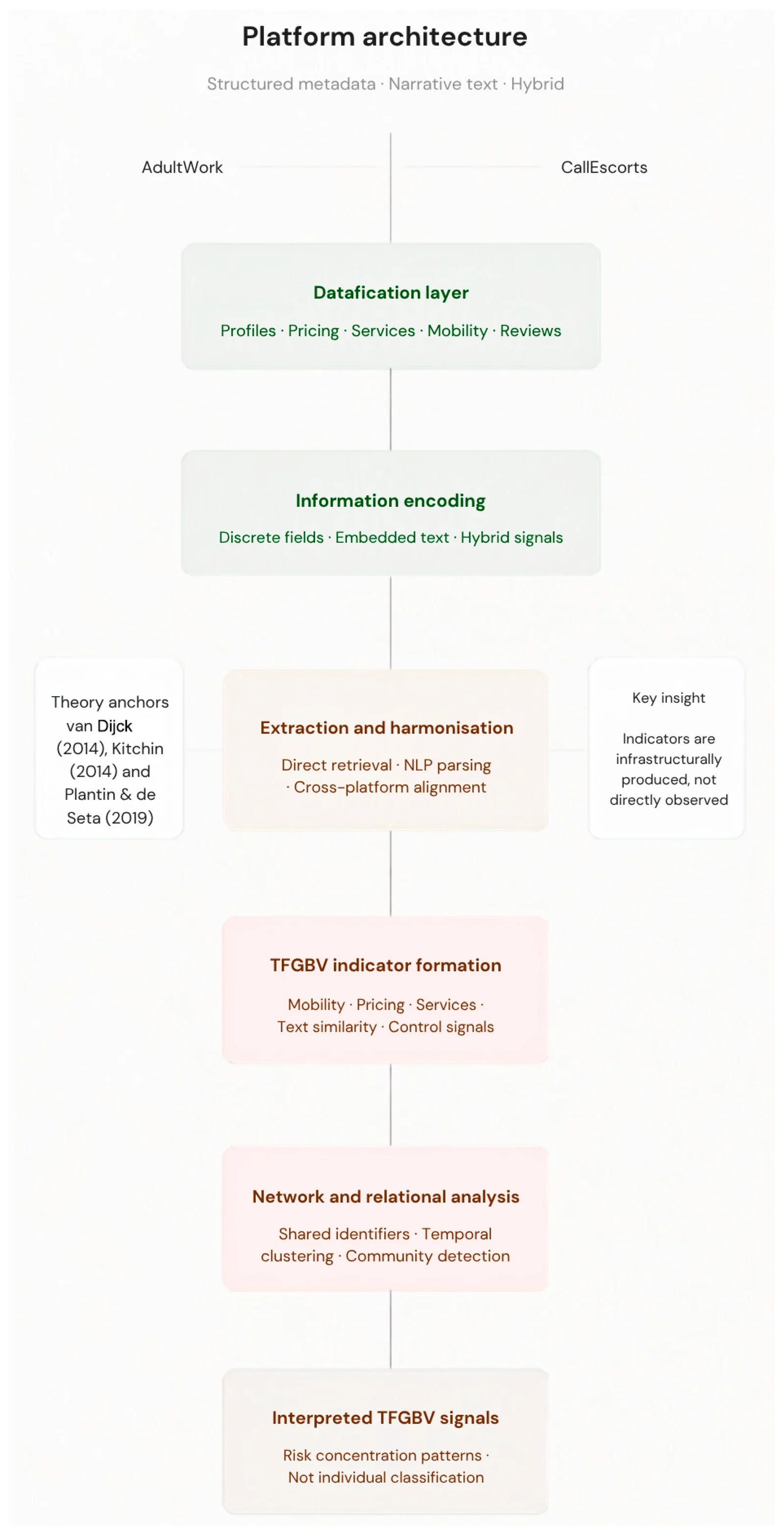
Platform-mediated visibility framework illustrating how platform architecture shapes the formation, extraction, and interpretation of potential exploitation-related indicators across AdultWork (UK) and CallEscort (US) (van Dijck, 2014; Kitchin, 2014; Plantin & de Seta, 2019).

The PMVF formalises this through five sequential stages, summarised in Table 4, and two additional steps adapted for the analysis conducted in this study (see the last two steps in Figure 1).

Stage 1 (Platform architecture) captures the fundamental design choice of whether user activity is encoded as structured metadata, unstructured narrative text, or some hybrid. This is the condition from which all subsequent variation follows. Stage 2 (Datafication layer) encompasses the digital traces platforms generate through user interaction; in this case, profiles, pricing records, service listings, client reviews, and location data. Stage 3 (Information encoding) describes how those traces are stored (e.g., as discrete machine-readable fields, embedded textual signals, or hybrid representations) and is the stage at which the two platforms examined here diverge most sharply. Stage 4 (Extraction and harmonisation) involves recovering analytically equivalent variables from structurally different encodings, requiring direct field retrieval in structured environments and NLP-based reconstruction in narrative ones, followed by cross-platform alignment to produce comparable indicators. Lastly, Stage 5 (Network and relational analysis) applies the harmonised indicators using analytical approaches based on the RQs. In the present study, this stage was operationalised through the identification of descriptive signs of activity (including shared phone numbers, duplicate descriptions, and geographic clustering) that may be relevant in the future to examining possible shared management, circuit mobility, or other coordinated activity previously considered in trafficking research. Such patterns do not independently establish third-party control, trafficking, or exploitation and may also have consensual or non-criminal explanations. Overall, the analytical stages show how, starting from platform architecture, comparisons allowed the identification of which variables may be relevant to potential exploitation-related indicators, although these considerations need to be cautiously interpreted (see Figure 1). The last two categories of the framework were included in Figure 1 (but not in Table 4) to display how the analysis was contextualised in this study. In other applications, these last steps can vary and be applied as relevant, with both indicators and interpretations being tailored accordingly.

The framework conceptualises visibility as the product of a series of platform-mediated processes through which behaviours become transformed into analysable data. Rather than treating indicators as objective features that exist independently within datasets, the PMVF positions them as outcomes of platform architecture, data representation, extraction processes, and analytical interpretation. In this context, visibility refers to the extent to which a behavioural phenomenon is represented in a form that can be systematically observed, extracted, and analysed using digital trace data. The findings of the present study provide empirical evidence of these processes within online commercial sex markets. Information relating to mobility, nationality, pricing, services, and profile activity existed across both platforms, but required subsequent levels of analysis to be linked from the initial differential representational mechanisms. The resulting indicators were therefore shaped by the infrastructures through which behaviours and variables were recorded.

This contribution has broader significance for TFGBV research. Scholarship in this field has reported that technologies are not neutral channels through which abuse occurs but socio-technical environments that shape how violence is enacted, experienced, and sustained. For example, Henry and Powell (2018) established that prevention requires engagement with the technological mediators of violence, not only with the individuals who perpetrate it. The present study operationalises this insight at the infrastructural level: if platform design determines what is observable, then responsible TFGBV research and prevention must account for both analytical methods and platform design. Analytical approaches developed using structured platforms may identify patterns invisible on narrative platforms; safeguarding tools calibrated to one jurisdiction’s dominant platform type may perform poorly in another’s. Regulatory frameworks that require platforms to structure certain information in machine-readable forms could improve the consistency and observability of relevant data but also increase the surveillance and exposure of consensual sex workers. Any such requirements would therefore need to be necessary, proportionate, privacy-preserving, and developed through engagement with affected communities. Additional recent work has started extending this argument to trafficking and exploitation, demonstrating how platform affordances influence the operation of coercive control and exploitation within digital environments (Stephenson et al., 2025). The present study advances these discussions by examining how platform infrastructures may shape the production of knowledge about exploitation. Researchers, practitioners, and law enforcement agencies encounter potential exploitation-related indicators within platform-generated data rather than direct evidence of exploitation. However, the role and impact of platform architecture are underplayed. The findings provide, as a result, an evidence base for specifying what information and what encoding standards matter most for analysis, while recognising the associated privacy and surveillance risks. As the UK Online Safety Act and the US *Kids online safety act* (2025, S. 1748) move toward greater platform accountability (Department for Science, Innovation & Technology, 2025), the PMVF may offer a methodical framework for evaluating how data architecture affects the visibility and responsible interpretation of potential TFGBV-related indicators.

The findings also contribute to an emerging body of research examining online commercial sex platforms as active mediators of visibility. Pajnik and Kuhar (2025) argue that platform affordances simultaneously create opportunities and constraints for self-presentation, disclosure, and professional practice within online sex work environments. The comparison between AdultWork and CallEscort supports this interpretation. Differences in platform architecture influenced the extent to which users disclosed demographic information, represented services, communicated pricing structures, and documented geographic mobility. These differences did not necessarily indicate behavioural variation, but alternative conditions under which behaviours became represented as data. Consequently, cross-platform comparisons that do not account for architectural differences risk conflating variations in representation with variations in behaviour. These observations have important implications for computational approaches to examining potential TFSE-related indicators. Existing studies have demonstrated the value of network-based and multi-indicator approaches that move beyond isolated profile characteristics (Dubrawski et al., 2015; Keskin et al., 2021; Summers et al., 2023). N. K. Freeman et al. (2022) similarly showed how digital trace data can support collaborative efforts between researchers and law enforcement agencies to identify networks potentially warranting further contextual assessment. Such approaches have been reported to outperform analyses based on individual indicators because they capture patterns of coordination, movement, and connectivity across accounts. The present findings support this, while adding that computational models remain dependent upon the visibility conditions embedded within platform architectures. Models trained within one platform environment may perform poorly when transferred elsewhere because the indicators available to them differ according to how information is structured and represented. The challenge extends, as a result, beyond algorithmic performance into data observability and comparability.

This challenge becomes increasingly important as computational approaches continue to expand within TFGBV research. Recent developments in machine learning and large language models have created new opportunities for analysing large-scale digital trace data, identifying behavioural patterns, and potentially informing safeguarding interventions (Summers et al., 2023; Lugo-Graulich et al., 2024). However, the effectiveness of these approaches remains dependent upon the quality and visibility of the underlying data. Similar concerns have been raised more broadly within TFGBV research, where questions of measurement, representativeness, and data quality remain central methodological challenges (University of Melbourne & United Nations Population Fund, 2023).

## 5. Limitations and Future Research

While this study introduces an analytical framework for cross-country platform comparisons and demonstrates how platform architecture shapes the visibility of potential exploitation-related indicators, several methodological and practical limitations should be acknowledged. First, the study does not have access to ground-truth data confirming whether profiles or networks were associated with human trafficking or TFSE. Consequently, the indicators examined should not be interpreted as definitive evidence of exploitation or as independently warranting investigation or intervention. They are potential indicators requiring contextual assessment and corroboration. Second, the study relies exclusively on publicly visible profile data, which excludes private communications, off-platform coordination, and encrypted interactions in which exploitation is known to occur (L’Hoiry et al., 2024). Potential indicators relating to control, coercion, and third-party management are, as a result, interpreted using public traces rather than direct observation. The study captures only the publicly observable profile activity. Third, the equalisation procedure aligned both platforms to the same seven-week window for the cross-sectional analytic sample; however, the raw datasets remain asymmetric in temporal depth (approximately seven weeks of AdultWork data versus four years for CallEscort), meaning that longitudinal analyses are possible only on the full CallEscort dataset. Future work should seek equivalent multi-year collection windows on both platforms to support valid cross-national comparison. The differences in datasets, despite the adjustments implemented, limit direct temporal comparison and mean that longitudinal patterns (see, for example, profile lifecycles, mobility, pricing changes over time) could only be observable in the US dataset. Future work should prioritise equivalent collection windows to support valid cross-national comparison. Fourth, the extraction of structured variables from CallEscort relied on NLP and rule-based parsing, which can introduce classification error, particularly for profiles using non-English language, coded terminology, or non-standard formatting. Validation of NLP extraction outputs against verified ground-truth cases, ideally with appropriate ethical oversight and input from law enforcement, sex workers, survivors, and relevant specialist organisations, is a priority for subsequent work.

Despite the limitations, the findings point towards several directions for future research that extend beyond methodological and detection refinement. A central implication of the PMVF, which is the novel contribution of this study, is that potential exploitation-related indicators may be examined as interconnected patterns whose visibility is shaped by platform architecture. As mentioned above, much of the existing trafficking detection literature has focused on identifying individual indicators such as mobility, pricing patterns, duplicated content, or references to youth (Dubrawski et al., 2015; Polaris Project, 2017). Nonetheless, emerging insights suggest that greater analytical value may lie in examining clusters of behaviours, temporal trajectories, and relational patterns that emerge across profiles, networks, and digital landscapes (Lugo-Graulich et al., 2024; Summers et al., 2023).

Work undertaken by organisations such as herEthical AI (n.d.), for instance, reflects a natural progression of this framework, moving beyond keyword- or indicator-based detection towards the identification of coercive control as a patterned phenomenon within narrative and behavioural data (Hall, 2024). Rather than treating coercive control as a single observable risk indicator, this approach integrates linguistic cues, relational dynamics, escalation patterns, and contextual indicators across large datasets, drawing from psychological research and evidence-based practice (Sayer et al., 2025).

## 6. Implications for Practice

The findings indicate that the identification of coordinated activity within online commercial sex platforms is shaped by the structure and availability of platform data. Although both platforms enabled the identification of descriptive activity-based factors, differences in profile architecture, metadata availability, and information standardisation influenced the extent to which behavioural relationships could be observed over time. The availability and structure of platform information, therefore, influenced the identification of behavioural patterns potentially relevant to TFSE research. The findings support the development of frameworks that consider platform infrastructure when assessing potential exploitation-related risks.

As discussed in Section 5, recent initiatives have moved beyond individual keywords or isolated indicators towards behavioural clusters and relational patterns. This reflects a growing recognition that potential indicators of exploitation are more likely to manifest through patterns of activity than through any single observable characteristic (Lugo-Graulich et al., 2024; Summers et al., 2023). In this study, the ability to identify such patterns varied according to the information preserved within each platform environment.

The implications extend to safeguarding and platform governance. Tools developed to support risk assessment and prevention, including emerging AI-assisted approaches, are necessarily constrained by the data available for analysis. Platform design decisions concerning metadata, profile structure, location disclosure, account persistence, and information standardisation affect the extent to which potential exploitation-related indicators can be identified. Platforms that facilitate consistent representation of information may support more effective analysis, whereas fragmented information structures may limit the identification of meaningful patterns. However, increasing the visibility and standardisation of sensitive information may also increase the risk of surveilling, exposing, or criminalising consensual sex workers. Greater visibility should therefore not be treated as inherently beneficial or as evidence of exploitation. This aligns with broader platform governance scholarship, which emphasises that infrastructural design decisions shape the social consequences of digital systems (Gillespie, 2018). For prevention and safeguarding, developing responsible analytical approaches therefore requires attention to both analytical methods and the digital infrastructures through which behavioural patterns are recorded. Any future operational application would require the safeguards outlined in Section 2.2.

## 7. Conclusions

The findings suggest that platform architecture functions as an epistemic filter through which potential exploitation-related indicators become observable within digital trace data. Existing trafficking detection frameworks have focused primarily on whether indicators can distinguish exploitation from consensual commercial sexual activity. The comparative US-UK analysis presented here demonstrates that an equally important question concerns whether those indicators are visible at all. The Platform-Mediated Visibility Framework responds to this challenge by positioning visibility as a product of platform architecture, data representation, extraction processes, and analytical interpretation. As such, the framework provides a foundation for future comparative research, responsible computational analysis, and evidence-informed approaches to understanding technology-facilitated sexual exploitation and the broader field of TFGBV.

## Figures and Tables

**Table 1:** Profile filtering outcomes by criterion.

Filtering Criterion	AdultWork Removed	CallEscort Removed
Incomplete: missing description and city (AdultWork)/title (CallEscort)	1541 (6.4%)	7935 (0.4%)
Duplicate description text (≥10 identical profiles)	933 (3.8%)	330,716 (14.6%)
Cam-service template text (AdultWork only)	779 (3.2%)	-
Shell accounts (0 images, 0 reviews, 0 services)	2977 (12.3%)	8890 (0.4%)
Platform-specific repeat contact criterion (≥3 profiles)	52 (0.2%)	1176 (0.1%)
Junk description (≤20 characters)	66 (0.3%)	25,466 (1.1%)
Total excluded (union of any criterion)	4774 (19.7%)	357,773 (15.8%)
Final analytic dataset before down sampling	19,488 (80.3%)	1,909,361 (84.2%)
Equalised analytic sample (post-equalisation)	17,061	17,061

**Table 2:** Descriptive characteristics of AdultWork (UK) and CallEscort (US) profile datasets.

Characteristic	AdultWork (UK) (*n* = 17,061)	CallEscort (US) (*n* = 17,061)
**Age (years)**		
Data available, *n* (% of *n*)	13,425 (78.7%)	17,055 (99.9%)
Mean (SD)	33.9 (9.7)	27.3 (5.9)
Median (range)	31 (18–80)	26 (18–75)
**Age group, *n* (% of those with age)**		
18–24	1758 (13.1%)	5897 (34.6%)
25–34	6343 (47.2%)	9232 (54.1%)
35–44	3247 (24.2%)	1529 (9.0%)
45–54	1494 (11.1%)	327 (1.9%)
55+	583 (4.3%)	70 (0.4%)
**Gender, *n* (% of *n*)**		
Woman	17,061 (100.0%)	Not recorded
**Sexual orientation, *n* (% of those with orientation)—AdultWork only; 52.8% of *n* available**		
Straight	3669 (40.7%)	-
Bi-sexual	2899 (32.2%)	-
Bi-curious	2428 (26.9%)	-
Not Specified	14 (0.2%)	-
Gay	6 (0.1%)	-
**Nationality, *n* (% of those with nationality)—AdultWork only; 52.8% of *n* available**		
British	5619 (62.4%)	-
Brazilian	1085 (12.0%)	-
Romanian	485 (5.4%)	-
Thai	381 (4.2%)	-
Polish	263 (2.9%)	-
Hungarian	192 (2.1%)	-
Other (108 nationalities)	980 (10.9%)	-
**Top cities, *n* (% of those with city)**		
City data available, *n* (% of *n*)	13,280 (77.8%)	17,061 (100.0%)
London (AW)	772 (5.8%)	-
Manchester (AW)	360 (2.7%)	-
Birmingham (AW)	238 (1.8%)	-
Atlanta (CE)	-	473 (2.8%)
Houston (CE)	-	469 (2.7%)
Dallas (CE)	-	425 (2.5%)
Chicago (CE)	-	360 (2.1%)

Note: Both samples are equalized to *n* = 17,061 advertisers drawn from the same 7-week window (10 February–31 March 2026), one row per advertiser. CallEscort was restricted to that window, deduplicated by phone number (latest scrape retained), then stratified-random subsampled by state × scrape-month (102 strata, proportional allocation, largest-remainder rounding, seed 564) to match the AdultWork *n*. AdultWork was restricted to women (*n* = 17,061), the closest available alignment because CallEscort does not record gender; the gender row is therefore 100% by design. Denominators differ by block and are stated in each section header: age-group percentages use the number of profiles with age recorded, not *n*; orientation, nationality and city percentages use the number with that field recorded. SD is the sample standard deviation. Hyphens indicate the platform does not record the field. Source: US-UK-analytic.sqlite (ads.analytic_sample = 1), built by build_analytic_sample.py on 17 August 2026.

**Table 3:** Cross-platform variable mapping: structural asymmetries and analytical implications.

AdultWork Variable	CallEscort Equivalent	Analytical Implications
services (list of strings)	Embedded in body text	No structured services field in CallEscort; identical information (e.g., service types) appears as free text in profile body. Recoverable via NLP extraction.
pricing.incall/pricing.outcall	Embedded in body text	Prices are sometimes listed in the body using shorthand conventions (e.g., ‘250QV’, ‘300HH’, ‘475HR’), but not as structured fields (as they are on AdultWork). Requires rule-based parsing.
metadata.gender	Implied by site category	Gender is not stored explicitly on CallEscort, but rather inferred from the site section placement (e.g., Female Escorts, TS/TG). Introduces classification uncertainty.
Interview: ‘Towns/Areas you will visit’	Cities worked (count)	Both capture geographic mobility. AdultWork stores as free-text list; CallEscort as a derived count variable. Structurally distinct but conceptually equivalent.
vital_statistics.Nationality	Occasionally in body text	Nationality is self-disclosed in the narrative (e.g., descriptive phrases), but there are no structured fields. Coverage is sparse and inconsistent.
Review count/review content	Not available	AdultWork stores structured client reviews linked to profiles. CallEscort does not provide equivalent review data, limiting longitudinal reputation analysis.

**Table 4:** The Platform-Mediated Visibility Framework: stages, definitions, and application.

Stage	Component	Definition	Application in This Study
1	Platform architecture	Structural design choices governing how user content is stored: structured metadata fields vs. narrative free-text vs. hybrid systems.	AdultWork: structured fields. CallEscort: narrative text. This determines baseline indicator visibility before any data collection begins.
2	Datafication layer	Digital traces generated through platform use: profiles, pricing records, service listings, client reviews, location data, timestamps.	Both platforms generate traces, but their form differs. AdultWork produces discrete records; CallEscort embeds equivalent information in text.
3	Information encoding	The specific form in which traces are stored: discrete machine-readable variables, embedded textual signals, or hybrid representations.	The critical divergence point. Indicators observable as variables in AdultWork are dispersed across unstructured text in CallEscort.
4	Extraction and harmonisation	Recovery and standardisation of indicators across platforms: direct field retrieval (structured) or NLP/rule-based reconstruction (narrative), followed by cross-platform alignment.	NLP, regular expression parsing, and entity extraction applied to CallEscort. Harmonised variables enable cross-platform comparison but introduce extraction errors.
5	Network and relational analysis	Application of harmonised indicators to identify descriptive patterns of activity: shared identifiers, geographic clustering, temporal co-occurrence, text similarity.	Shared identifiers, duplicate descriptions, and city-movement patterns analysed across both platforms. Signals were treated as probabilistic rather than predictive.

## Data Availability

The datasets generated and analyzed during the current study are not publicly available due to ethical and legal considerations relating to the source platforms from which the data was collected.

## Note

1 The legal contrast is one of degree rather than kind. In England and Wales, selling and purchasing sex are not themselves offences, but a range of surrounding activities are, including brothel-keeping, soliciting in a public place, and controlling prostitution for gain or paying for the sexual services of a person subjected to force. Northern Ireland departs from this position by criminalising the purchase of sexual services. In the United States, prostitution is a criminal offence in every state except for licensed brothels operating in rural Nevada counties.
